# White Matter Changes With Rehabilitation in Children With Developmental Coordination Disorder: A Randomized Controlled Trial

**DOI:** 10.3389/fnhum.2021.673003

**Published:** 2021-06-03

**Authors:** Sara Izadi-Najafabadi, Jill G. Zwicker

**Affiliations:** ^1^Graduate Programs in Rehabilitation Sciences, University of British Columbia, Vancouver, BC, Canada; ^2^Brain, Behaviour, and Development Theme, BC Children’s Hospital Research Institute, Vancouver, BC, Canada; ^3^Department of Occupational Science and Occupational Therapy, University of British Columbia, Vancouver, BC, Canada; ^4^Department of Pediatrics, University of British Columbia, Vancouver, BC, Canada; ^5^Sunny Hill Health Centre at BC Children’s Hospital, Vancouver, BC, Canada; ^6^CanChild Centre for Childhood Disability Research, Hamilton, ON, Canada

**Keywords:** developmental coordination disorder, motor skills disorder, rehabilitation, diffusion tensor imaging, neuroplasticity, CO-OP

## Abstract

**Background and Objectives**: Children with developmental coordination disorder (DCD) have difficulty learning motor skills, which can affect their participation in activities of daily living and psychosocial well-being. Over 50% of children with DCD also have attention deficit hyperactivity disorder (ADHD), which further exacerbates their motor problems and impact on quality of life. A rehabilitation approach known as Cognitive Orientation to Occupational Performance uses problem-solving strategies to help children learn motor skills they wish to achieve. While this cognitive approach has been effective for children with DCD, few studies have examined the effectiveness of this approach for children with co-occurring ADHD. Further, the underlying mechanism and neural basis of this intervention are largely unknown.

**Methods**: In this randomized waitlist-controlled trial, we used MRI to examine white matter microstructure after intervention in 8–12-year-old children with DCD (*n* = 28) and with DCD and co-occurring ADHD (*n* = 25). Children in both groups were randomized to either a treatment group or waitlist group at their first MRI. The treatment group began the intervention after their MRI scan and returned for a post-treatment scan at 3 months, and follow-up scan at 6 months; the waitlist group waited 3 months before their second MRI, received the intervention, and then had a post-treatment scan. Each child received intervention once weekly for 10 weeks. Diffusion tensor imaging was used to acquire white matter diffusion parameters and was analyzed using tract-based spatial statistics (TBSS).

**Results and Conclusion**: Children with DCD showed significant improvement in white matter microstructure in the bilateral anterior thalamic radiation, bilateral sensorimotor tract, bilateral cingulum, fornix, splenium and body of corpus callosum, right inferior fronto-occipital fasciculus, and white matter pathways to bilateral inferior gyri, right middle frontal gyrus, frontal medial cortex, and left cuneus. We suggest that these rehabilitation-induced neural changes in children with DCD occurred in regions associated with attention, self-regulation, motor planning, and inter-hemispheric communication, which positively affected brain connectivity and motor function. In contrast, children with DCD and co-occurring ADHD did not show any brain changes following the intervention. Modifications to the treatment protocol might help address the attentional and self-regulatory needs of children with a dual diagnosis.

**Clinical Trial Registration**: ClinicalTrials.gov ID: NCT02597751.

## Introduction

Up to 5 to 6% of all school-age children may be affected by developmental coordination disorder (DCD), a neurodevelopmental disorder characterized by difficulty performing and learning motor skills that significantly interferes with daily activities and academic achievement (American Psychiatric Association, 2013). These motor difficulties can lead to higher risks of anxiety, emotional and behavioral problems, low self-esteem (Hellgren et al., 1994; Green et al., 2006; Pratt and Hill, 2011; Lingam et al., 2012; Hill and Brown, 2013; Zwicker et al., 2013; Crane et al., 2017; Harrowell et al., 2017; Li et al., 2018), as well as physical consequences such as obesity and poorer physical fitness (Cairney et al., 2005, 2010; Rivilis et al., 2011; Cairney and Veldhuizen, 2013). A well-known and common co-occurrence of DCD is attention deficit hyperactivity disorder (ADHD), exacerbating motor and functional difficulties in children (Kadesjo and Gillberg, 1999; Piek et al., 1999; Dewey et al., 2000, 2002; Rasmussen and Gillberg, 2000; Pitcher et al., 2003; Martin et al., 2006; Watemberg et al., 2007; Fliers et al., 2009; Barkley, 2014; Blank et al., 2019) and increasing the risk of psychological distress (Piek et al., 2007; Missiuna et al., 2014), antisocial behavior (Rasmussen and Gillberg, 2000), and peer victimization (Dewey and Volkovinskaia, 2018). Motor problems of children with DCD with or without ADHD have been attributed to attention deficits and lack of inhibition (Kaiser et al., 2015; Fong et al., 2016; Thornton et al., 2018). An electroencephalographic (EEG) study suggests that the contribution of attention to motor performance is greater in children with co-occurring DCD and ADHD than children with DCD only (Fong et al., 2016); it, accordingly assumes that improving attention in children with DCD with or without ADHD leads to motor performance improvement (Fong et al., 2016). However, only 30% to 50% of children with DCD and ADHD show improved motor performance following attention-related medications (Bart et al., 2010; Brossard-Racine et al., 2012), and only 50% of children with ADHD receive non-pharmaceutical treatment for their motor difficulties (Fliers et al., 2010, 2011). This controversy in the literature and limited attention to motor problems add complexities to the treatment approaches for children with a dual diagnosis of DCD and ADHD.

Current neuroimaging studies reveal that DCD is associated with involvement of the cerebellum, the parietal lobe, the frontal lobe, the basal ganglia, and the limbic system (Brown-Lum and Zwicker, 2015; Biotteau et al., 2016); each of these regions play a specific role in generating internal models of motor actions (Kawato and Gomi, 1992; Blakemore et al., 2001), updating the internal model (Blakemore and Sirigu, 2003), providing optimal control during motor execution (Shadmehr and Krakauer, 2008), executing motor actions (Shadmehr and Krakauer, 2008), and managing the movement motivation (Merel et al., 2019), respectively.

However, co-occurring DCD and ADHD are associated with unique structural (Langevin et al., 2014, 2015), functional (McLeod et al., 2014, 2016; Thornton et al., 2018), and physiological (Yeh et al., 2012; Fong et al., 2016) properties of the sensorimotor and attentional networks, including the parietal and frontal lobes (Yeh et al., 2012; Langevin et al., 2014, 2015; McLeod et al., 2014, 2016; Thornton et al., 2018) as well as interhemispheric connections and asymmetry (Langevin et al., 2014, 2015). Individuals with co-occurring DCD and ADHD may use compensatory attentional control of motor coordination through increasing cerebral blood flow in the posterior cingulate cortex and the cerebellum (Yeh et al., 2012).

A treatment approach called Cognitive Orientation to daily Occupational Performance (CO-OP) is one of the recommended treatments for DCD as per international clinical guidelines (Blank et al., 2019). CO-OP is a client-centered, task-oriented approach developed for children with DCD to successfully solve motor problems (Polatajko et al., 2001). Previous studies have shown positive results in children with DCD (Ward and Rodger, 2004; Taylor et al., 2007; Zwicker et al., 2015; Capistran and Martini, 2016; Thornton et al., 2016), but given that at least 50% of children with DCD have co-occurring ADHD (Dewey et al., 2002), we wondered if this cognitive approach was effective for children with a dual diagnosis of DCD and ADHD. Results from a single case study of six children with ADHD show promise for the CO-OP approach (Gharebaghy et al., 2015), but studies with larger sample sizes and more rigorous research designs are required to determine CO-OP’s effectiveness in children with DCD, with and without ADHD. To better understand if and how CO-OP affects children with DCD with or without ADHD differently, we examined brain changes after 10 sessions of CO-OP intervention using various MRI modalities (resting state, T1-weighted images, and diffusion tensor imaging; DTI). In the current study, we focus on structural neuroplastic changes captured by DTI after CO-OP intervention in children with DCD, with and without ADHD. Understanding the neural mechanisms of CO-OP could further guide the modification and optimization of CO-OP based on specific needs of children with DCD with or without ADHD.

Diffusion MRI is a non-invasive tool measuring both structural connectivity and white matter microstructure by obtaining information about connections between brain regions and their tissue architecture (Jones et al., 2013). It measures water diffusivity in brain tissue and the amount of restriction experienced by water molecules moving in the brain. Water molecules are considerably impeded in white matter, owing to factors such as myelination, fiber diameter or density, as well as membrane permeability (Beaulieu, 2002). This impedance causes directional and anisotropic water diffusivity.

Thus far, diffusion MRI studies have used various analysis methods, including tract-based spatial statistics (TBSS; Williams et al., 2017; Brown-Lum et al., 2020), constrained spherical deconvolution (Hyde et al., 2019), tractography (Zwicker et al., 2012; Debrabant et al., 2016) and graph theory (Debrabant et al., 2016) to understand white matter microstructure in children with DCD. Results have shown that children with DCD have altered white matter microstructure in the corpus callosum (Langevin et al., 2014; Brown-Lum et al., 2020) and sensorimotor, corticospinal, cortico-cerebellar (Zwicker et al., 2012; Debrabant et al., 2016; Williams et al., 2017; Brown-Lum et al., 2020), and frontoparietal pathways (Langevin et al., 2014; Williams et al., 2017; Hyde et al., 2019; Brown-Lum et al., 2020). Structural connectivity between brain regions (e.g., cerebellar lobule VI and right superior parietal gyrus; Debrabant et al., 2016) is also implicated in children with DCD. Children with DCD and co-occurring ADHD have altered white matter in the corpus callosum (Langevin et al., 2014). However, no study investigated longitudinal changes following intervention in children with DCD. In this study, we will compare white matter microstructural properties of children with DCD, with or without ADHD, before and after CO-OP intervention.

## Materials and Methods

### Study Design

In this randomized waitlist-controlled trial (ClinicalTrials.gov ID: NCT02597751), we used computer-generated sequential blocks of four to six, prepared by a statistician to randomize children with DCD, with or without ADHD, into treatment and waitlist groups. To ensure a power of 90% to detect a 3% difference in axial diffusivity (AD) with a type-1 error of 0.01, we used our pilot study on DTI in this population (effect size = 1.1; Zwicker et al., 2012) and estimated a sample size of 27 per group.

### Participants

From September 2014 to July 2019, children with DCD and DCD+ADHD were recruited from the Sunny Hill Health Centre for Children, BC Children’s Hospital ADHD Clinic, the Vancouver Regional Pediatric Team, and from advertisements in the community (Vancouver, BC, Canada). Children aged 8–12 years were eligible to participate in the study if they were diagnosed with DCD as per the Diagnostic and Statistical Manual–5th edition (American Psychiatric Association, 2013) and the international clinical practice recommendations (Blank et al., 2019) as follows: (1): scored ≤16th percentile on the Movement Assessment Battery for Children—2nd ed. (MABC-2; Henderson et al., 2007); (2) scored in the suspected or indicative DCD range on the DCD Questionnaire (Wilson et al., 2009); (3) parents reported motor difficulties from a young age; and (4) there was no other medical condition that could explain motor difficulties based on parent-report, clinical observations, and/or a medical examination. Children were assigned to the DCD+ADHD group if they had been diagnosed ADHD based on parent report. ADHD symptomatology was quantified for all children using the Conners ADHD Index—parent report form (Conners, 2009). Exclusion criteria included premature birth (gestational age <37 weeks), other neurodevelopmental disorders (e.g., autism spectrum disorder), claustrophobia, and MRI contraindications (e.g., metal braces). After parental consent and child assent as per ethics approval from the University of British Columbia/BC Children’s and Women’s Research Ethics Board, children were enrolled in the study.

### Procedure

Participants started with MRI safety screening and an MRI simulation session. A research nurse accompanied by a graduate student scanned all the participants using MRI at baseline, after 3 months, and after 6 months. After the first MRI scan, children were randomized into treatment and waitlist groups; group assignments were concealed to the research team in an opaque, sealed envelope. Children assigned to the treatment group went through pre-intervention assessment, then received 10 sessions of CO-OP intervention (once a week), followed by post-intervention assessment, a second (post-treatment) MRI scan, and a third follow-up MRI scan 3 months later to determine if brain changes were maintained after the intervention was discontinued. In contrast, children in the waitlist group were scanned 3 months after their first MRI, received their pre-assessments and intervention, followed by a third (post-treatment) MRI. A study design schematic (Izadi-Najafabadi et al., 2020) can be found elsewhere.

Motor assessments were administered by an occupational therapist not involved in the intervention. Assessments included the following: (1) Canadian Occupational Performance Measure (COPM; Law et al., 2014), which measured the child’s perceived motor performance and satisfaction on their three chosen motor goals; (2) Performance Quality Rating Scale (PQRS; Martini et al., 2015), which objectively measured the child’s quality of motor performance on their motor goals based on a blinded occupational therapist scoring videos of the child performing their motor goals before and after the intervention; and (3) Bruininks-Oseretsky Test of Motor Proficiency-2 (BOT-2) short form (Bruininks and Bruininks, 2005), which measured general motor skills. A more detailed description of behavioral outcome measures and their results can be found elsewhere (Izadi-Najafabadi et al., under review).

### Intervention

Using the Pediatric Activity Card Sort (Mandich et al., 2004), an assessment tool for developing activity profiles for children, each child with DCD (with or without ADHD) identified three functional motor goals (e.g., handwriting, playing basketball, tying shoelaces) on which to work during the CO-OP intervention. An occupational therapist administered 10 one-hour sessions of the CO-OP intervention over 10 weeks as per published protocol (Polatajko et al., 2001) at the Sunny Hill Health Centre for Children or BC Children’s Hospital. CO-OP intervention is a problem-solving rehabilitation approach that focuses on the use of cognitive strategies to solve motor problems. Children learned the global strategy of CO-OP called Goal-Plan-Do-Check in the first session and were guided to discover specific cognitive strategies (e.g., supplementing task knowledge, changing body position) to solve motor problems to achieve their chosen goals (Miller et al., 2001; Polatajko and Mandich, 2004). Parents were instructed and encouraged to use CO-OP at home and keep a record of practice time for each goal on each day, per week.

### Diffusion Tensor Imaging

#### Acquisition

A 3-Tesla General-Electric Discovery MR750 MRI scanner with a 32-channel head coil was used to acquire DTI data. Participants were asked to lie very still during the DTI sequence while watching a movie. A minimum of two 32-direction DTI sequences were acquired so that the best sequence could be used for data analysis. DTI acquisition parameters for each DTI sequence with 32 diffusion encoded directions dispersed around a full-sphere were as follows: TR: 7,000 ms; TE: 60 ms; FOV: 220 mm; acquisition matrix: 100 × 100; slice thickness: 2.2 mm; *b* = 1,000. Three *b*0 volumes were also acquired at the beginning of each DTI sequence. A graduate student monitored movement during the scan, encouraged the child to stay still, and asked the sequence to be repeated, if needed.

#### Preprocessing

DTI data preprocessing and analysis were completed using FSL 6.0.1. (Smith et al., 2004). Preprocessing steps included: (1) executing *eddy_cuda* for distortion correction through signal loss (drop-out) detection and replacement (Andersson et al., 2016) as well as correction for susceptibility-induced distortions, eddy currents, and subject motions (within and between volumes; Andersson and Sotiropoulos, 2016; Andersson et al., 2017); (2) implementing automated quality control via *QUality* *Assessment for DMRI (QUAD)* to extract quality metrices of within-and between-volume average relative motion, absolute relative motion, signal-to-noise ratio (SNR), and contrast-to-noise ratio (CNR; Bastiani et al., 2019); for each participant, the sequence with higher CNR was carried forward for further analysis; (3) visually checking every image to identify any residual distortion and removing any motion-contaminated volumes; any image with less than 20 good quality volumes were excluded from the analysis; and (4) reconstructing diffusion tensors to estimate DTI parameters and create corresponding maps: *fractional anisotropy (FA)*, the degree of anisotropy/directionality of water diffusion in each voxel; *mean diffusivity (MD)*, average amount of water diffusion independent of directionality in each voxel; *axial diffusivity (AD)*, water diffusivity along the tract; *radial diffusivity (RD)*, water diffusivity perpendicular to the tract (Jones et al., 2013).

#### Statistical Analysis

We performed tract-based spatial statistics (TBSS) to analyze whole-brain white matter microstructural properties without pre-specification of tracts of interest (Smith et al., 2006). TBSS carries out a voxel-wise statistical analysis while controlling for inaccurate alignment and arbitrary smoothing in traditional voxel-based analysis (Smith et al., 2006). TBSS aligns each participant’s FA map to the FA map of the most representative participant and then to the standard template (MNI 152 1 mm). The use of the most representative participant as the first step in registration is critical to TBSS reliability (Madhyastha et al., 2014) since it increases the alignment accuracy and reduces inter-subject variability (Smith et al., 2006). A mean FA skeleton was then generated using all participants’ aligned FA maps and thresholded at 0.2 to exclude any gray matter residuals and peripheral tracts with high inter-subject variability. Each participant’s FA, MD, AD, and RD data were then projected onto the mean FA skeleton to be ready for statistical analysis (Smith et al., 2006). TBSS has shown a high test-retest reliability in longitudinal studies (Madhyastha et al., 2014).

TBSS results were fed into Permutation Analysis of Linear Models (PALM; Winkler et al., 2014) with 5,000 permutations to investigate 3-month maturation effect (scan 1 and 2 of waitlist groups), pre-post intervention effect (scan 1 and 2 of treatment group combined with scan 2 and 3 of waitlist group), and 3-month follow-up effect (scan 2 and 3 of treatment group) for children with DCD and DCD+ADHD. We used a paired *t*-test design controlling for ADHD-related medications. Family-wise error correction (FWE) with an alpha level of 0.05 was applied to correct for multiple testing errors. We also used PALM to run a generalized linear model and investigate the effect of motor outcomes (PQRS; Martini et al., 2015) and BOT-2 (Bruininks and Bruininks, 2005) on DTI parameters in the two groups. To report the results, we used a sensitive thresholding approach called threshold-free cluster enhancement (TFCE), in which voxel-wise values receive local spatial supports from extended areas of signal (Smith and Nichols, 2009). To label anatomic locations of white matter structures, the Johns Hopkins University ICBM-81 White-Matter Labels (Mori et al., 2005), the White Matter Tractography Atlas (Hua et al., 2008), and the Sensorimotor Tracts Atlas (Archer et al., 2018) were used.

## Results

### Participants

**Figure 1:**
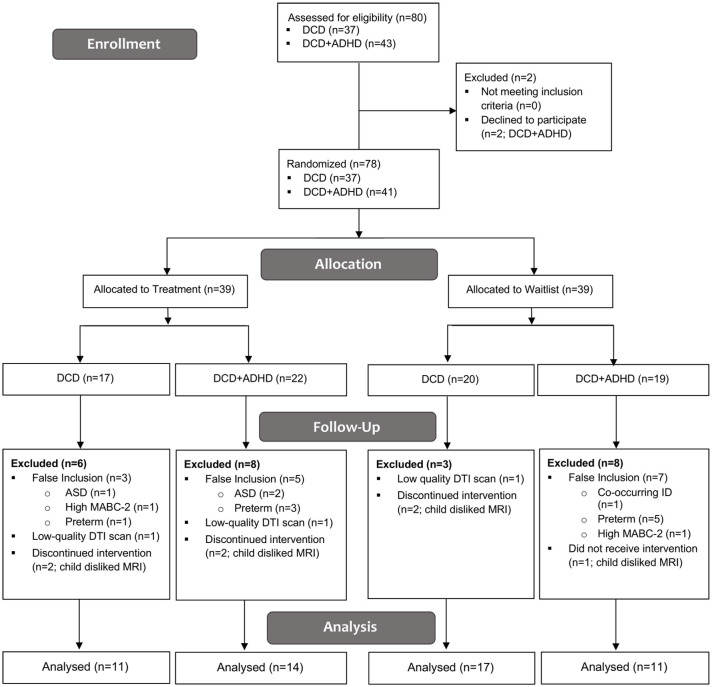
CONSORT Flow Diagram. ADHD, attention deficit hyperactivity disorder; ASD, autism spectrum disorder; DCD, developmental coordination disorder; DTI, diffusion tensor imaging; ID, intellectual disability; MABC-2, Movement Assessment Battery for Children—2nd ed.

Eighty children were recruited for this study; two children with DCD + ADHD declined to participate. Of the 78 children enrolled and randomized into treatment and waitlist groups, 37 children were diagnosed with DCD [25 male, 12 female; mean (SD) age: 9.7 (1.5) years] and 41 children were diagnosed with both DCD and ADHD [38 male, 3 female; mean (SD) age: 10.2 (1.4) years]. Nine children with DCD and 16 children with DCD + ADHD were excluded from the analysis due to false inclusion (DCD: *n* = 3; DCD + ADHD: *n* = 12), low MRI quality (DCD: *n* = 2; DCD + ADHD: *n* = 1), and child disliked MRI (DCD: *n* = 4; DCD + ADHD: *n* = 3). See Figure 1 for more details.

Accordingly, we compared pre-and post-intervention scans from 28 children with DCD [20 male, 8 female; mean (SD) age: 9.6 (1.4) years] and 25 children with DCD + ADHD [22 male, 3 female; mean (SD) age: 9.9 (1.1) years]. Due to the quality of baseline and follow-up scans, maturation effect (DCD: *n* = 18; DCD + ADHD: *n* = 10) and follow-up analyses (DCD: *n* = 10; DCD + ADHD: *n* = 13) were performed with fewer participants. At the time of intervention, three children with DCD and 13 children with DCD + ADHD took ADHD-related medications (e.g., Adderall, Biphentin, Concerta). Table 1 summarizes participant characteristics and Table 2 highlights DTI quality and head motion parameters per group.

**Table 1:** Participant characteristics.

Variable	DCD (*n* = 28)		DCD + ADHD (*n* = 25)	
	Treatment (*n* = 11)	Waitlist (*n* = 17)	Treatment (*n* = 14)	Waitlist (*n* = 11)
Male sex; N (%)	7 (64)	13 (77)	12 (86)	10 (91)
Age (years); Mean (SD)	10.1 (1.6)	9.3 (1.2)	9.7 (1.2)	10.2 (1.1)
DCDQ (total); Mean (SD)	28.1 (8.6)	32.8 (9.8)	28.1 (6.7)	32.0 (6.8)
MABC-2 (percentile); Median (IQR)	2 (6.2)	2 (4.0)	1 (1.5)	9 (6.0)
Conner’s ADHD Index (T-score); Median (IQR)	90 (17)	86 (31)	90 (0)	90 (0)

*ADHD, attention deficit hyperactivity disorder; DCD, developmental coordination disorder; DCDQ, Developmental Coordination Disorder Questionnaire; IQR, inter-quartile range; MABC-2, Movement Assessment Battery for Children—2nd ed.; SD, standard deviation*.

**Table 2:** DTI quality and head motion parameters.

Variable		DCD (*n* = 28)		DCD + ADHD (*n* = 25)	
		Treatment (*n* = 11)	Waitlist (*n* = 17)	Treatment (*n* = 14)	Waitlist (*n* = 11)
**DTI Quality**					
Contrast-to-Noise Ratio (CNR); Mean (SD)	Scan 1	5.7 (1.0)	4.9 (1.7)	5.8 (1.8)	*10.2 (8.5)
	Scan 2	5.9 (1.2)	5.3 (2.0)	6.8 (4.1)	7.0 (3.6)
	Scan 3	5.6 (0.9)	5.8 (2.5)	5.5 (1.9)	6.6 (2.6)

Signal-to-Noise Ratio (SNR); Mean (SD)	Scan 1	65.6 (10.0)	61.0 (11.8)	65.1 (10.7)	61.4 (19.9)
	Scan 2	68.6 (8.0)	67.0 (13.2)	63.9 (10.0)	63.9 (14.8)
	Scan 3	71.7 (6.6)	65.8 (10.8)	65.8 (11.9)	72.4 (7.2)
**Head Motion Parameters**					
Average Relative Motion (mm); Mean (SD)	Scan 1	0.30 (0.2)	0.42 (0.2)	0.44 (0.2)	0.40 (0.3)
	Scan 2	0.20 (0.1)	0.45 (0.2)	0.41 (0.3)	0.40 (0.3)
	Scan 3	0.30 (0.2)	0.30 (0.2)	0.45 (0.3)	0.32 (0.13)

Average Absolute Motion (mm); Mean (SD)	Scan 1	0.81 (0.5)	1.20 (1.0)	1.20 (1.0)	1.10 (1.4)
	Scan 2	0.83 (0.7)	1.00 (0.5)	0.86 (0.6)	0.80 (0.4)
	Scan 3	0.73 (0.5)	0.80 (0.3)	1.10 (0.9)	0.90 (0.5)

ADHD, attention deficit hyperactivity disorder; DCD, developmental coordination disorder; DTI, diffusion tensor imaging; SD, standard deviation. *p < 0.01: not significant after Bonferroni correction (*p* < 0.004).

### DCD-Only Group

#### Maturation

**Figure 2:**
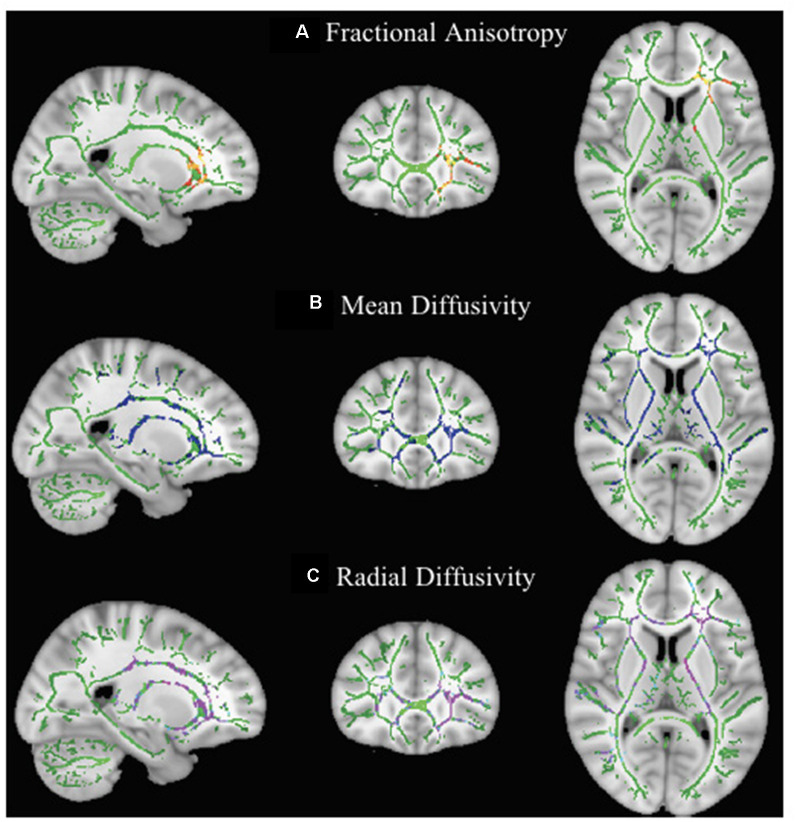
Effect of maturation on DTI parameters in children with DCD. In these images, the white matter skeleton is shown in green and brain structures with altered fractional anisotropy (FA; **A**), mean diffusivity (MD; **B**), and radial diffusivity (RD; **C**) are shown in red, blue, and purple, respectively.

Comparison of scan 1 and scan 2 of children in the waitlist group showed a significant decrease in FA and a significant increase in MD and RD of the left anterior thalamic radiation passing through the anterior corona radiata (FA: FWE-*p* < 0.05; MD and RD: FWE-*p* < 0.01) and anterior limb of internal capsule (FA and MD: FWE-*p* < 0.05; RD: FWE-*p* < 0.01) as well as a significant increase in MD and RD (FWE-*p* < 0.01) of the right anterior thalamic radiation, bilateral corticospinal tract, bilateral inferior longitudinal fasciculus, bilateral superior longitudinal fasciculus, bilateral inferior fronto-occipital fasciculus, bilateral cingulum, corpus callosum, and right anterior corona radiata, posterior limb of internal capsule, and retrolenticular part of internal capsule (Table 3 and Figure 2).

**Table 3:** Effect of maturation on DTI parameters in children with DCD^a^.

White matter structure		MNI-space			DTI	Direction	*t*	FWE	Cohen’s *d*
		*X*	*Y*	*Z*	parameter	of change		Sig	
Anterior thalamic radiation L	At anterior corona radiata	−21	31	8	FA	Decreased	3.9	*	0.72
		−24	32	9	MD	Increased	2.5	**	0.46
					RD		4.1	**	0.76
	At anterior limb of internal capsule	−20	17	4	FA	Decreased	3.0	*	0.55
		−12	2	4	MD	Increased	1.3	*	0.24
					RD		3.0	**	0.55
	At posterior limb of internal capsule	−12	−2	6	MD	Increased	2.8	**	0.52
					RD		2.2	**	0.41
	At thalamus	−10	−10	10	MD	Increased	1.5	*	0.27
Anterior thalamic radiation R		25	−35	4	MD	Increased	2.4	**	0.44
					RD		2.2	*	0.40
	At anterior corona radiata	22	31	10	MD	Increased	1.6	*	0.29
					RD		1.9	**	0.36
	At anterior limb of internal capsule	13	1	5	MD	Increased	4.9	**	0.88
					RD		4.6	**	0.66
Sensorimotor tract L	At posterior corona radiata	−25	−25	29	MD	Increased	2.5	*	0.46
					RD		3.8	**	0.70
Sensorimotor tract R	At posterior limb of internal capsule	18	−12	1	MD	Increased	2.9	**	0.54
					RD		2.2	*	0.40
	At thalamus	16	−20	0	MD	Increased	2.9	**	0.48
					RD		2.1	**	0.49
	At pontine crossing	6	−30	−35	MD	Increased	3.9	*	0.71
Inferior fronto-occipital fasciculus L		−23	23	6	MD	Increased	1.9	**	0.35
					RD		2.9	**	0.53
Inferior fronto-occipital fasciculus R		25	23	−7	MD	Increased	1.9	**	0.34
					RD		2.1	**	0.38
		33	−30	5	MD	Increased	3.2	**	0.59
					RD		1.1	*	0.20
Superior longitudinal fasciculus L		−36	−22	29	MD	Increased	1.4	*	0.25
					RD		4.1	**	0.75
Superior longitudinal fasciculus R		39	−36	34	MD	Increased	2.7	*	0.48
					RD		2.2	*	0.40
		31	−53	24	MD	Increased	1.2	*	0.23
					RD		3.4	*	0.62
		43	−46	3	MD	Increased	0.9	*	0.20
					RD		2.2	*	040
		58	−30	−14	MD	Increased	2.8	**	0.51
					RD		3.5	**	0.65
Inferior longitudinal fasciculus L		−29	−30	3	MD	Increased	1.4	*	0.25
					RD		2.4	**	0.43
		−43	−30	−7	MD	Increased	2.2	*	0.40
		−48	−21	−15			3.2	*	0.60
		−43	0	−30			2.8	*	0.50
		−45	−3	−34			1.9	*	0.30
Inferior longitudinal fasciculus R		43	−7	−20	MD	Increased	2.4	**	0.44
					RD		1.9	**	0.36
		48	116	60	MD	Increased	2.4	**	0.43
					RD		3.2	**	0.58
		42	−3	−24	MD	Increased	3.5	**	0.64
					RD		1.9	**	0.34
		45	−15	−10	MD	Increased	2.7	**	0.49
					RD		3.1	**	0.57
		43	−41	−3	MD	Increased	1.9	*	0.34
					RD		2.4	**	0.43
		40	−30	4	MD	Increased	1.9	**	0.34
					RD		4.4	**	0.81
Cingulum L		−8	−25	38	MD	Increased	1.7	*	0.31
					RD		2.0	*	0.37
		−18	−37	38	MD	Increased	2.9	*	0.52
					RD		3.6	*	0.65
Cingulum R		8	−25	38	MD	Increased	2.2	*	0.40
					RD		4.0	*	0.74
		10	−36	37	MD	Increased	2.3	*	0.40
					RD		1.8	*	0.33
Anterior corona radiata R		18	28	−10	MD	Increased	2.1	**	0.40
					RD		1.0	*	0.20
		27	17	18	MD	Increased	1.6	**	0.30
					RD		1.2	*	0.20
Posterior limb of internal capsule R		21	−5	13	MD	Increased	2.5	**	0.46
					RD		5.7	*	0.99
Retrolenticular of internal capsule R		28	−21	−3	MD	Increased	4.2	**	0.76
					RD		4.3	**	0.79
Corpus callosum	Genu	13	32	0	MD	Increased	1.1	**	0.30
Forceps minor		−18	54	6	RD	Increased	2.1	*	0.39
Body		4	12	21	MD	Increased	2.2	**	0.41
					RD		1.9	*	0.35
		0	3	24	MD	Increased	1.5	**	0.27
					RD		1.5	*	0.28
	Splenium	−2	−32	18	MD	Increased	2.3	**	0.43
					RD		1.6	*	0.30
		13	−43	13	MD	Increased	1.8	**	0.32

DCD, developmental coordination disorder; DTI, diffusion tensor imaging; FA, fractional anisotropy; FWE, family-wise error corrected; L, left; MD, mean diffusivity; R, right; RD, radial diffusivity; Sig, significant level. ^a^Effects are shown with threshold-free cluster enhancement (TFCE) and a minimum cluster size of 100 voxels. *FWE-*p* < 0.05; confidence interval: 0.044–0.056. **FWE-*p* < 0.01; confidence interval: 0.008–0.013.

#### Intervention Effect

**Figure 3:**
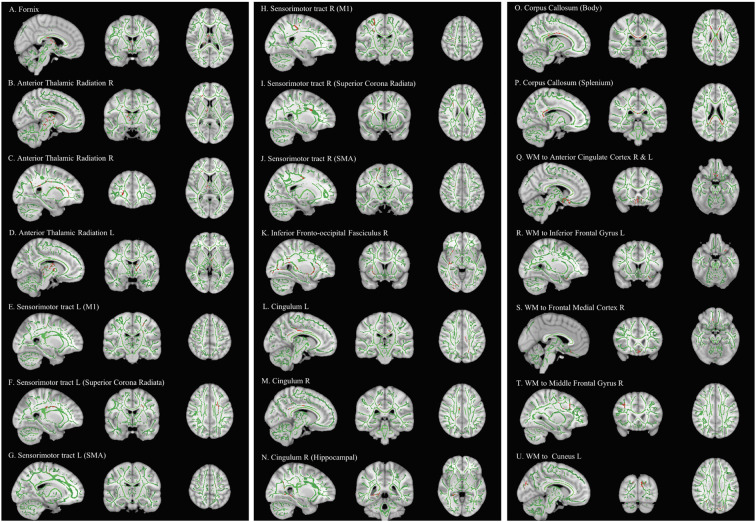
Effect of intervention on fractional anisotropy in children with DCD. In these images, the white matter skeleton is shown in green and brain structures with altered fractional anisotropy are shown in red. L, left; M1, primary motor cortex; R, right; SMA, supplementary motor area; WM, white matter.

Comparing pre-and post-intervention DTI parameters of 28 children with DCD showed a significant increase (FWE-*p* < 0.05) in FA of the bilateral anterior thalamic radiation, bilateral sensorimotor tract, bilateral cingulum, fornix, splenium and body of corpus callosum, right inferior fronto-occipital fasciculus, and white matter pathways to the bilateral inferior gyri, right middle frontal gyrus, frontal medial cortex, and left cuneus (Table 4 and Figure 3).

**Table 4:** Increased FA after the CO-OP intervention in children with DCD^a^.

White matter structure		MNI-space			*t*	FWE Sig	Cohen’s *d*
		*X*	*Y*	*Z*			
Fornix		0	−11	7	2.4	*	0.34
Anterior thalamic radiation L	At precuneus	−19	−55	38	1.3	*	0.18
	At posterior limb of internal capsule	−10	−2	6	4.8	*	0.68
	At red nucleus	−2	−21	−10	3.1	*	0.43
	At parahippocampal gyrus	−21	−34	1	2.9	*	0.41
	At thalamus	−8	−20	3	1.3	*	0.18
Anterior thalamic radiation R	At anterior nucleus	11	−4	10	2.3	*	0.33
	At precuneus	19	−55	38	3.9	*	0.56
	At parahippocampal gyrus	22	−36	3	2.0	*	0.28
	At thalamus	8	−20	3	1.0	*	0.14
Sensorimotor tract L	At M1	−24	−19	46	1.6	*	0.22
	At superior corona radiata	−24	−21	33	1.5	*	0.21
	At SMA	−16	−11	53	1.3	*	0.18
Sensorimotor tract R	At M1	24	−28	47	31.4	*	0.20
	At superior corona radiata	26	−18	34	2.5	*	0.35
	At SMA	12	−12	65	2.2	*	0.31
Inferior fronto-occipital fasciculus R		31	−66	1	5.1	*	0.72
	At cuneus	18	−84	22	1.2	*	0.17
	At external capsule	36	−10	−5	1.8	*	0.27
	At occipital pole	20	−93	2	3.1	*	0.43
	At tapetum	30	−44	17	3.1	*	0.43
Cingulum L	At posterior cingulate cortex	−13	−34	37	3.2	*	0.45
	At posterior corona radiata	−20	−30	35	3.2	*	0.45
	At subgenual anterior cingulate cortex	−6	15	−12	2.6	*	0.37
Cingulum R	At posterior cingulate cortex	13	−34	37	1.2	*	0.17
	At posterior corona radiata	20	−30	34	1.6	*	0.22
	At sub-genual anterior cingulate cortex	6	15	−12	1.5	*	0.22
Corpus callosum	Body	9	−7	28	1.2	*	0.17
	Body	−9	−7	28	2.4	*	0.34
	Body to cingulate cortex	−12	12	25	3.4	*	0.48
	Splenium	−25	−57	16	3.4	*	0.48
	Splenium to cingulate cortex	−12	−35	24	3.7	*	0.52
White matter	To subcallosal area of anterior cingulate cortex	7	21	−19	2.6	*	0.37
	To inferior frontal gyrus L	−32	18	−21	3.5	*	0.49
	To frontal medial cortex R	6	36	−23	1.9	*	0.27
	To middle frontal cortex R	32	23	32	1.4	*	0.21
	To cuneus L	−8	−86	29	3.4	*	0.48

DCD, developmental coordination disorder; FA, fractional anisotropy; FWE, family-wise error corrected; L, left; M1: primary motor cortex; R, right; Sig, significant level; SMA: supplementary motor area. ^a^Effects are shown with threshold-free cluster enhancement (TFCE) and a minimum cluster size of 100 voxels. *FWE-*p* < 0.05; confidence interval: 0.044–0.056.

#### Follow-up

Follow-up analysis of 10 children with DCD in the treatment group did not show any significant changes between post-intervention and follow-up MRI scans. We also did not find any significant changes from pre-intervention to follow-up scans in this small sub-sample.

#### Motor Outcomes

Regression analysis did not show any relationship between behavioral outcome measures (e.g., PQRS and BOT-2) and changes in white matter microstructure in children with DCD.

### DCD + ADHD Group

Children with DCD + ADHD did not show any significant change (FWE-*p* > 0.05) in any of the DTI parameters in the 3-month period before CO-OP intervention (*n* = 10), immediately after the intervention (*n* = 25), or in the follow-up scan (*n* = 13). We did not find any relationship between motor performance and white matter parameters.

## Discussion

Our results showed an increase in FA in white matter tracts, not associated with maturation, after CO-OP intervention in children with DCD, including the bilateral anterior thalamic radiation, bilateral sensorimotor tracts, right inferior fronto-occipital fasciculus, bilateral cingulum, fornix, and corpus callosum. During the first 3 months of the study prior to the CO-OP intervention, we observed an overall increase in RD and MD of the white matter microstructure as well as a reduction in FA of left anterior thalamic radiation in children with DCD. We showed that the CO-OP intervention reverted this pattern in children with DCD. Similar to our resting state results (Rinat et al., 2020), children with DCD + ADHD did not show any brain changes after the CO-OP intervention.

### Rehabilitation-Induced Neuroplasticity in Children With DCD

Improved FA in white matter tracts is generally thought to reflect improved microstructural properties, such as axonal density and diameter, myelin integrity, or fiber coherence in these tracts (Concha, 2014); however, the specific change in microstructure cannot be identified. It is also important to note that FA values can be imprecise in regions where there are crossing fibers (O’Donnell and Westin, 2011). Notwithstanding these limitations, we showed that FA increased in the bilateral anterior thalamic radiations, bilateral sensorimotor tracts, right inferior fronto-occipital fasciculus, bilateral cingulum, fornix, corpus callosum, and white matter structures to anterior cingulate cortex and frontal lobe after CO-OP intervention in children with DCD. Our results align with preliminary results from a non-parametric combination of voxel-based morphometry and fractional anisotropy in the anterior thalamic radiation and the body and splenium of the corpus callosum after the CO-OP intervention (Izadi-Najafabadi et al., 2020). These regions play multiple roles in human behavior (e.g., emotion regulation, motor control, executive function). In what follows, we will explain how improved FA in each of these white matter tracts is related to the CO-OP intervention and its potential role in self-regulation, attention, and emotion regulation, as well as motor performance.

### Attention and Self-regulation

Consistent with behavioral evidence of self-regulation mediating CO-OP’s effect (Jokić et al., 2013), our results suggest that CO-OP intervention might play a role in self- and attention-regulation. Most of our findings underlie the default mode network (DMN); *anterior thalamic radiation, cingulum, corpus callosum, inferior fronto-occipital fasciculus*, and *white matter tracts to the cuneus and* *anterior cingulate cortex* connect DMN regions in the posterior and anterior cingulate cortices, thalamus, cuneus, as well as across the two hemispheres (Teipel et al., 2010; Luo et al., 2012; Alves et al., 2019). DMN is involved in internally-directed attention, regulating attentional resources, and guiding self-regulatory processes (Bush et al., 2000; Grimm et al., 2009; Kelly et al., 2009; Wiebking et al., 2011; Dixon et al., 2014). The CO-OP intervention uses a global strategy of *Goal-Plan-Do-Check* where children set goals and motivate themselves, perform the task, observe their own performance, judge it to improve it, and then achieve their goal and express their self-satisfaction (Polatajko and Mandich, 2004), which is similar to the three phases of self-regulation (i.e., forethought, performance, and self-reflection) based on Triadic Model of Self-regulation (Zimmerman, 2000).

We have also shown that CO-OP intervention induces both increased FA in white matter structures underlying the DMN and increased functional connectivity between the DMN and the anterior cingulate cortex in this population (Rinat et al., 2020). High functional connectivity between regions of the DMN is related to their increased structural connectivity (van den Heuvel et al., 2008; Damoiseaux and Greicius, 2009), in particular, increased FA in the right and left cingulum (Bathelt et al., 2019).

Moreover, CO-OP intervention induces structural changes in other DMN white matter tracts, including the corona radiata (Izadi-Najafabadi et al., 2020). Based on our results from DTI analysis and other analysis (Izadi-Najafabadi et al., 2020; Rinat et al., 2020), we infer that the CO-OP intervention improves both functional and structural connectivity of the DMN, which is responsible for attention-and self-regulation (Shamloo and Helie, 2016; Reddy et al., 2018; Izadi-Najafabadi et al., 2020). We have previously suggested that these functions are implicated in children with DCD due to altered connectivity between the posterior cingulate cortex and DMN (Rinat et al., 2020).

CO-OP intervention improved white matter organization of the *right inferior fronto-occipital fasciculus*, *right anterior thalamic radiation, and bilateral sensorimotor tracts*. These regions are involved in various forms of attention-and self-regulation. The *inferior fronto-occipital fasciculus* guides sustained attention and other executive control functions (Leng et al., 2016). It is the only tract with a rightward asymmetry in our result, which is consistent with the right asymmetry for sustained attention (Pardo et al., 1991). Brown-Lum (2017) reported reduced FA in the *right inferior fronto-occipital fasciculus* in children with DCD. The white matter organization of the *right inferior fronto-occipital fasciculus* and *the right anterior thalamic radiation* are also associated with delayed discounting—the ability to tolerate delays while waiting for future rewards (Olson et al., 2009). Delayed discounting is a critical aspect of motivated behavior and self-regulatory skills, which are known to mediate the CO-OP intervention effects in children with DCD (Jokić et al., 2013).

The *bilateral sensorimotor tracts* immediately beneath the superior corona radiata transfer motor information from the primary motor cortex (M1) and supplementary motor area (SMA) through the corticospinal tract (Klöppel et al., 2008; Vergani et al., 2014). High FA in the superior corona radiata is also associated with improved focused and sustained attention (Stave et al., 2017) and has previously been reported in a different analysis of CO-OP-induced structural changes (Izadi-Najafabadi et al., 2020). The current analysis provides further evidence of the potential role of the CO-OP in attention regulation and motor execution.

### Emotion Regulation

We found improved microstructure in regions involved in emotion regulation including *the anterior thalamic radiation, SMA white matter, and inferior frontal gyrus white matter*. Emotion regulation is a process of modifying emotion to increase or decrease emotional experience before, during, and after the motor performance (Beatty and Janelle, 2020). Accordingly, we suggest that the CO-OP intervention might help to regulate emotions required before, during, and after motor learning; the child-chosen nature of motor goals in CO-OP might increase emotional motivation prior to the motor performance, which is known to be the action starter (Beatty and Janelle, 2020). To achieve motor goals, CO-OP enables children to use strategies that distract them from negative emotions (e.g., frustration or embarrassment of their inability to perform a task) during motor skill acquisition. For example, when children use the domain-specific strategy of “attention to doing” during CO-OP, they focus more on their action rather than their feeling. Lastly, when children achieve their motor goals, they experience satisfaction; all these together enhance the likelihood of success in future motor performance and helps with emotion regulation (Beatty and Janelle, 2020). Moreover, similar to a key feature of CO-OP (Missiuna et al., 2001), emotions could be regulated when guided by a significant adult (e.g., therapist or parents; Williams et al., 2020).

The *anterior thalamic radiation* is known for its role in emotional processes and low FA in this structure is related to sadness (Coenen et al., 2012; Jia et al., 2014; Niida et al., 2018). Anterior parts of the *cingulum*, terminating in the frontal lobe, are also involved in attention and executive function (Takahashi et al., 2010; Chiang et al., 2016). The *anterior thalamic radiation* along with the *cingulum* are considered parts of the Papez circuit, involved in emotional processing, semantic memory, and learning (Papez, 1937; Jang and Yeo, 2013; Bubb et al., 2017; Weininger et al., 2019). Extremely high or low levels of inputs from the anterior thalamic nuclei to the anterior cingulate cortex through the *anterior thalamic radiation* and the *cingulum* could increase emotional distraction of behavior (Hartikainen et al., 2014; Sun et al., 2015) and overwhelm attentional resources required for learning (Öhman et al., 2001; Hartikainen et al., 2014). As such, we infer that CO-OP intervention might regulate emotions by allocating more attentional resources to motor performance, which is reflected in neuroplastic changes in white matter microstructure. This interpretation should be considered with caution considering that we did not assess emotion and attention regulation before and after the intervention.

FA in the *white matter tract to the right inferior frontal gyrus* is involved in executive functions, detection of cues (Hampshire et al., 2010), and fine movement control (Liakakis et al., 2011). The *inferior frontal gyrus* has structural connectivity with the *SMA* (Kohn et al., 2014; Vergani et al., 2014), which mediates overt and covert speech initiation (Winsler et al., 2009), as a means of emotion regulation (Beatty and Janelle, 2020). CO-OP uses self-verbalization in the form of overt and covert/inner speech production as a global strategy to solve motor problems (Missiuna et al., 2001) and children’s verbal ability predicts their motor outcomes after the CO-OP (Green et al., 2008). Self-verbalization strategies of CO-OP (e.g., global strategy of Goal-Plan-Do-Check and domain-specific strategies of verbal motor mnemonic and verbal rote script; Polatajko and Mandich, 2004) may motivate children by providing effective instructions and selectively attending to the performance-relevant information rather than negative emotions (Anderson, 1997; Beatty and Janelle, 2020). Self-verbalization helps to internalize instructions and use them to acquire the skills (Anderson, 1997). Accordingly, the observed increase in FA of white matter structures underlying the SMA and inferior frontal gyrus might be associated with CO-OP’s extensive use of self-verbalization strategy as a regulatory technique.

The SMA also plays a role in planning and executing a motor function. It has structural connectivity with the *cingulate gyrus* (Vergani et al., 2014), transforming emotion into motor experience in situations of reward or punishments (Northoff et al., 2000; Oliveri et al., 2003; Kohn et al., 2014). Taken together, our findings suggest that the CO-OP intervention may play a role in emotion regulation as well. More research is required to better understand CO-OP’s role in emotion regulation at behavioral and neural levels.

### Motor Performance

We also found increased FA in the *bilateral sensorimotor tracts* immediately beneath the right and left M1, as well as left SMA and parts of the right and left superior corona radiata. Since we did not perform tractography, we are not able to distinguish between corticospinal tracts and corticobulbar tracts originating from sensorimotor cortical areas. In looking at regional microstructure, increased regional FA in the subcortical white matter areas could be explained in an intra-hemispheric or inter-hemispheric context. As for intra-hemispheric improvement, high regional FA underneath M1 might indicate high functional connectivity between M1 and secondary motor areas in each hemisphere (Klöppel et al., 2008; Vergani et al., 2014), especially in the left hemisphere lateralized for motor control (Guye et al., 2003). However, our resting state results did not show such increased functional connectivity (Izadi-Najafabadi et al., 2020). This discrepancy between our structural and functional results might have different explanations. First, it could be due to a smaller sample size in our resting state analysis or a false positive result in our DTI results. Also, considering the small effect size in the sensorimotor tracts (Cohen’s *D* range: 0.18–0.35), these results should be interpreted with caution. Second, it could be related to the unique role of brain function and structure in supporting a specific behavior (Zimmermann et al., 2018). Although structural and functional connectivity are equally important in understanding behavior, they capture independent and complementary features of the brain and do not necessarily show spatial overlap (Zimmermann et al., 2018). Further studies are required to better understand the relationship between brain function/ structure and motor behavior in children with DCD.

Increased FA in the *bilateral sensorimotor tracts* immediately beneath M1 and SMA may be indicative of improved structural connectivity between motor-related brain regions. A transcranial magnetic stimulation study showed that water diffuses from M1 to the subcortical white matter below the prefrontal areas, as well as parts of the corona radiata, internal capsule, cerebral peduncles, and corpus callosum (Klöppel et al., 2008). Also, the SMA sends fibers through the corpus callosum and through the corona radiata to join the corticospinal tract (Vergani et al., 2014). Similarly, we found increased FA in the subcortical white matter immediately underneath M1, SMA, middle and inferior frontal gyrus, frontal medial cortex, as well as parts of the superior corona radiata and corpus callosum. Therefore, we suggest that CO-OP intervention increases FA in the subcortical white matter below motor cortices, facilitates cortico-cortical connectivity between M1 and SMA and/or corticospinal connectivity; this can subsequently improve planning, initiating, and executing a motor task. This interpretation should be considered with caution and further analysis using tractography and graph theory is required to better understand the nature of increased FA in these subcortical white matter structures.

As for inter-hemispheric improvement in FA, right and left M1 and SMA are connected through transcallosal sensorimotor fibers that facilitate interhemispheric inhibition (Fling et al., 2013; Vergani et al., 2014). FA of these transcallosal sensorimotor fibers at the superior portion of the corticospinal tracts are positively correlated to motor ability of children as assessed by MABC-2 (Grohs et al., 2018). Motor learning, especially during bimanual movements, is known to enhance inter-hemispheric interactions (Takeuchi et al., 2012); in our study, all children had chosen at least one bimanual motor goal, such as tying shoelaces or cutting a fruit with a knife, which might explain the improved microstructure of transcallosal sensorimotor fibers.

We did not find any relationship between motor performance and microstructural changes while we had previously reported a relationship between improved motor performance and functional connectivity of the cerebellar lobule I-IV and the DMN related to automatization (Izadi-Najafabadi et al., 2020). Evidence suggests that the brain-behavior relationship is explained differently when considering brain function vs. brain structure. In a large study investigating the whole-brain structural connectivity-behavior relationship vs. functional connectivity-behavior, there were fewer structural connections that were linked to specific behaviors than functional connectivity. And, unlike functional connectivity, a general pattern of structural connectivity is linked to overall cognitive abilities (Zimmermann et al., 2018). These findings may explain why we found a functional connectivity-motor relationship in our results while we did not find such a specific relationship using our microstructural measures. Moreover, we suggested that CO-OP was associated with improved microstructural properties of regions involved in self-regulation, attention regulation, and emotion regulation; accordingly, we may not have found a brain-behavior relationship because we did not assess these mediatory behaviors.

### Theories to Support Interpretation of Findings

Our results and interpretation of the role of self-regulation, attention, and emotion regulation on motor performance are consistent with the “sensorimotor control framework for emotion regulation” by Williams et al. (2020) and the “Optimizing Performance through Intrinsic Motivation and Attention for Learning (OPTIMAL) theory of motor learning" by Wulf and Lewthwaite (2016).

The sensorimotor control framework for emotion regulation is similar to the internal modeling hypothesis (Adams et al., 2014), with an added component of emotion. Accordingly, all actions start with a motivation or emotional value. To achieve this desired goal, children have to form internal sensorimotor models over time and to learn and plan their movements based on feedback-feedforward processes (Williams et al., 2020). Children may achieve their goal or fail, which engages their emotional response again and modulates their actions; strategies such as avoiding, selective attention, and reappraisal are used to regulate emotions until the child achieves the goal (Braunstein et al., 2017). The OPTIMAL theory of motor learning emphasizes the role of emotions and motivation in motor performance (Wulf and Lewthwaite, 2016). This theory predicts a virtuous vs. a vicious cycle in motor learning; in the virtuous cycle, motivation leads to better motor performance, a sense of autonomy, and self-efficacy, which in turn, motivates the individual to further accomplish motor goals. The vicious cycle, on the other hand, predicts that low motivation results in low motor performance and* vice versa* (Wulf and Lewthwaite, 2016).

### Atypical Maturation in Children With DCD

The pattern of brain maturation prior to the intervention in children with DCD contradicts existing reports of longitudinal development in typically developing children and adolescents; typically, white matter microstructure develops with increased FA and decreased MD and RD (Chen et al., 2016; Krogsrud et al., 2016). The observed reduction in FA and increase in MA and RD in this study suggests an overall decline in brain microstructure in children with DCD over a 3-month period. Reduced FA in the left anterior thalamic radiation in children with DCD compared to typically developing children has previously been reported (Brown-Lum, 2017). The current study is the first to report a longitudinal decrease in FA of this white matter structure in children with DCD, suggesting atypical brain development/maturation might underlie their motor performance difficulty.

In a different analysis, we were not able to find any maturational changes in white matter FA and/or volume in a sample of 12 children with DCD +/– ADHD (Izadi-Najafabadi et al., 2020). The smaller sample size may have reduced the statistical power to detect brain changes, and the inclusion of children with DCD + ADHD could have confounded the results.

In addition, we were not able to find any maturational changes in the white matter microstructure of children with DCD + ADHD prior to the intervention, indicating that their brain development does not follow a typical trajectory. This finding should be interpreted with caution as this might be due to a smaller sample size (DCD: *n* = 18; DCD + ADHD: *n* = 10) and reduced statistical power.

### No Observed Neuroplasticity in Children With DCD + ADHD

Although CO-OP improved the motor performance of children with DCD + ADHD (Izadi-Najafabadi et al., under review), they did not show any changes in white matter microstructure after the intervention. This is in line with Green et al.’s (2008) study indicating the benefits of CO-OP for children with co-occurring conditions such as ADHD while still experiencing motor problems. Our results are explained considering three evidence-informed statements: (1) the CO-OP intervention relies on attention and self-regulation to mediate its effects on motor performance (Jokić et al., 2013); (2) attention contribution to motor performance is greater in children with DCD + ADHD than children with DCD only (Fong et al., 2016); (3) children with ADHD experience greater difficulty with self-regulation (e.g., attention regulation, emotion regulation; Shiels and Hawk, 2010). In other words, the CO-OP intervention is not able to overcome the attentional and self-regulatory difficulties and its underlying neural mechanism in children with DCD + ADHD. Moreover, children with DCD + ADHD have shown unique brain structure (Langevin et al., 2014, 2015) and function (McLeod et al., 2014, 2016; Thornton et al., 2018) compared to children with DCD. Compensatory attentional control by the posterior cingulate cortex and the cerebellum has been proposed as the neural mechanism of motor coordination in children with DCD + ADHD (Yeh et al., 2012); however, our resting state (Izadi-Najafabadi et al., in press), morphometry (Izadi-Najafabadi et al., 2020), and diffusion imaging analyses did not show similar neural mechanism following the CO-OP.

Additionally, literature suggests that only hyperactivity symptoms of children with ADHD improve over time, and this improvement is highly correlated with reduced FA in the left corticospinal tract (Francx et al., 2015); it is, therefore, hypothesized that development induces a less stimulated/excited motor pathway with decreased FA, which reduces the motor hyperactivity in children with ADHD (Francx et al., 2015). On the other hand, reduced FA in white matter microstructure over time, especially in the frontal and parietal regions, could reflect poorer motor performance as seen in children with DCD (Brown-Lum et al., 2020) and DCD + ADHD (Langevin et al., 2014). Thus, it is unclear whether a reduced FA in the corticospinal tract is beneficial or detrimental in children with ADHD. This highlights how a co-occurring condition such as ADHD complicates our understanding of brain and neuroplasticity following the intervention.

Taken together, some modifications to the CO-OP protocol to better address the attentional needs of children with ADHD (Gharebaghy et al., 2015; Izadi-Najafabadi et al., 2020) or combining CO-OP intervention with medication or other self-regulatory interventions (Reid et al., 2005) might improve its effectiveness and induce permanent brain changes in children with DCD + ADHD.

## Limitations

A smaller than anticipated sample size for the analysis could have biased our results. This study is the first study to use DTI to investigate training-induced brain changes in children with DCD; however, we only performed a voxel-wise analysis of the white matter microstructure, which has spatial inaccuracy (Edden and Jones, 2011). TBSS assumes an accurate registration of the data and it is nearly impossible to detect errors (Jones and Cercignani, 2010); this could add some uncertainty to the results. Further analysis using myelin water fraction analysis, tractography (probabilistic or constrained spherical deconvolution), and graph theory would help to validate our results. In addition, although we conducted the strictest available clean-up pipeline through FSL and excluded any residual motion-contaminated volumes from our data, we did not collect field map data, which would have further cleaned our data and better corrected for the susceptibility-induced distortions. Moreover, we did not find a relationship between behavioral data and white matter microstructure, meaning that behavioral interpretations should be considered with caution.

## Conclusions

Our results indicate that the CO-OP intervention induces microstructural changes in white matter tracts involved in self-, attention-, and emotion-regulation and white matter structures involved in intra-and inter-hemispheric transfer of motor information. These changes were maintained 3 months after the intervention. However, children with a dual diagnosis of DCD and ADHD did not show any microstructural changes following CO-OP intervention, suggesting their different needs in motor-based interventions. These results are consistent with our functional connectivity results of improved motor performance and associated increases in functional connectivity of DMN with the pACC after CO-OP intervention in children with DCD, but not in children with a dual diagnosis of DCD and ADHD (Izadi-Najafabadi et al., 2020).

## Data Availability Statement

The raw data supporting the conclusions of this article will be made available by the authors, without undue reservation.

## Ethics Statement

The studies involving human participants were reviewed and approved by University of British Columbia/BC Children’s and Women’s Research Ethics Board. Written informed consent to participate in this study was provided by the participants’ parent/legal guardian. Child assent was also obtained.

## Author Contributions

SI was responsible for participant screening, data collection, data processing, statistical data analysis, data interpretation, and drafting the manuscript. JZ conceived and designed the study, coordinated and supervised data collection, and critically reviewed the draft manuscript for intellectual content. All authors contributed to the article and approved the submitted version.

## Conflict of Interest

The authors declare that the research was conducted in the absence of any commercial or financial relationships that could be construed as a potential conflict of interest.
